# The severity of depression is associated with pelvic inflammatory diseases: A cross-sectional study of the United States National Health and Nutrition Examinations from 2013 to 2018

**DOI:** 10.3389/fmed.2022.926351

**Published:** 2022-10-12

**Authors:** TianJiao Huang, RenShuang Cao, PengFei Liu, JinXing Liu, Xiao Yu

**Affiliations:** ^1^First College of Clinical Medicine, Shandong University of Traditional Chinese Medicine, Jinan, China; ^2^College of Traditional Chinese Medicine, Shandong University of Traditional Chinese Medicine, Jinan, China; ^3^Department of Gynecology, Affiliated Hospital of Shandong University of Traditional Chinese Medicine, Jinan, China

**Keywords:** pelvic inflammatory diseases, NHANES, depression severity, cross-sectional study, inflammatory state

## Abstract

**Purpose:**

As depression in patients with pelvic inflammatory diseases (PID) has received increasing attention in recent years, this study aims to investigate the relationship between depression severity and risk factors for pelvic inflammatory disease, and to provide new perspectives in the treatment of PID.

**Patients and methods:**

Multivariate regression was used to evaluate the association between pelvic inflammatory disease and the severity of depression. Females who participated in the United States National Health and Nutrition Examination Survey (NHANES) from 2013 to 2018 were included. In addition, risk factors for PID and depression were also included in the analysis as adjustment factors.

**Results:**

The risk of developing PID was associated with depressive status (odds ratio, OR 1.10, 95% confidence interval, CI 1.08–1.12), especially in people with severe depression (odds ratio, OR 6.34, 95% confidence interval, CI 3.72–10.79). Subgroup analysis showed differences in the risk of PID among people with different characteristics.

**Conclusion:**

This study showed that there may be a potential positive association between depressive status and the prevalence of PID in the United States adult female population. Depression should be actively looked for in all patients with PID and treated appropriately

## Introduction

Pelvic inflammatory disease is a common clinical syndrome of female reproductive tract, which is usually caused by untreated pathogenic microorganism infection, which may lead to important reproductive sequelae, including tubal infertility, chronic pelvic pain, ectopic pregnancy and so on (1). The etiology of pelvic inflammatory diseases (PID)is complicated (2, 3). Anaerobes, viruses, and mycoplasma are also involved in sexually transmitted infections (4). The prevalence of sexually transmitted infection-related diseases in high-income countries is estimated at 40% due to PID (5). Clinical signs and symptoms do not clearly indicate PID, and some women of childbearing age show no signs of infected reproductive organs, which severely affects their physical and mental health (1).

Research indicates that inflammation plays a role in the pathophysiology of depression. Inflammation, or inflammatory reaction, is caused by the activation of the immune system, usually characterized by local reactions caused by stimulation, injury, or infection, accompanied by symptoms such as redness, swelling, heat, and pain (6). Redness, swelling, heat, and pain were commonly associated with local reactions caused by stimulation, injury, or infection (7). In patients with severe depression, the abnormal expression of glucocorticoid receptors is related to inflammation (8). Furthermore, patients with depression are at risk for congenital and adaptive immune system disorders, etc. Inflammation is conducive to a decreased antidepressant response to depression (9). Most PID can be treated with antibiotics. Nonetheless, the therapeutic effect is not optimal because of drug resistance and side effects (10). There is limited clinical evidence on depression states and inflammatory disorders such pelvic inflammatory disease, despite a huge corpus of research on inflammation levels and depressive states.

The United States (US) National Health and Nutrition Survey (NHANES)is one of several health-related programs conducted by the NCHS to inform the public on the health and nutritional status of diseases and conditions as well as provide information on the prevention of the diseases or conditions. Health policy decisions were made using this information to estimate the prevalence of various diseases and conditions (11). The NHANES database has many perfect and standardized physiological and psychological data such as depression index questionnaire and medical history collection. Considering that depression is closely related to inflammatory factors, the relevant data of NHANES database are included in the research and analysis. The study aims to determine whether depression is associated with the prevalence of PID to shed light on new ideas for the clinical treatment of PID.

## Materials and methods

### Study population

We used data on NHANES participants from 2013 to 2018 for this study. Female participants aged 12 years and older were eligible. The Research Ethics Review Committee of the National Center for Health Statistics (NCHS) approved the NHANES project, and the data in NHANES were collected by professional investigators of NCHS. No application is required to use the database and it is available to any researcher who meets the requirements for use. All patient information in the database is anonymous, and all participants are aware of and consent to the data collection activities.

### Primary exposure

The Patient Health Questionnaire of NHANES database, which consists of nine items, was administered for depression screening. “Almost every day,” “a few days,” “more than half of the days,” and “not at all” were scored 0–3 according to the nine-item instrument. The total score is calculated by adding up the scores in each item, ranging from 0 to 27. PHQ-9 scores and Proposed Treatment Actions were used to define the rating criteria for depression, depression is classified according to the score of depression index:None (0–4), Mild (5–9), Moderate (10–14), Moderately Severe (15–19), Severe (20–27).

### Outcomes

Prevalence of PID was assessed based on the NHANES Reproductive Health Questionnaire, where RHQ078 asked, “Ever treated for a pelvic infection/PID?” Participants who answered “Yes” were categorized as the PID group.

### Covariates

NHANES investigators prespecified options for race/ethnicity based on a written questionnaire. Another included a multiracial option. Race/ethnicity and Education level were included in the analysis because it is known to be independently associated with PID. According to guidelines published by the US Centers for Disease Control and Prevention, a z score for age categorizes body mass index as normal. Whether there was a regular menstrual cycle in the past 12 months, and the number of leukocytes and neutrophils in serum were taken from NHANES questionnaire data and laboratory data, respectively, and were included in the study as risk factors for PID events.

### Statistical analysis

Continuous variables were expressed as means and standard deviations in normal distributions or as medians (quartiles) in skewed distributions. Among the measurements, we used a one-way ANOVA (normal distribution), K-W test (skewed distribution) and chi-square test (categorical variables) to assess whether there was any significant difference between means and proportions.

Multivariate logistic regression analysis and curve fitting was used to assess the association of the primary outcome: prevalence of PID, with the Depression Severity. By adjusting different risk factors step by step, we tested different models (Models 1–3). Model 1 was Unadjusted, Model 2 was adjusted for age, education, and race; Model 3 was further adjusted for Regular menstruation and BMI; Based on these data, an adjusted odds ratio (OR) is calculated with 95% confidence intervals (CI). The strengthening the reporting of observational studies in epidemiology (STROBE) statement advised simultaneous presentation of results from unadjusted, minimally adjusted, and fully adjusted analyses. The covariance in the model adjusts at least 10% of the matched OR values. All analyses were carried out using R (^1^ The R Foundation) and EmpowerStats (X&Y Solutions, Inc., Boston, MA, USA). Statistical significance was defined as *P* values less than 0.05 (two-sided).

### Participant characteristics

**FIGURE 1:**
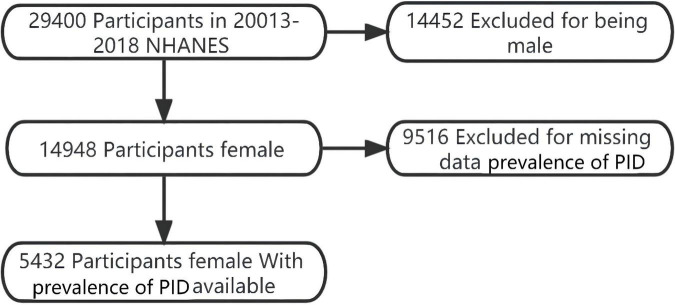
Flowchart of study participant selection: from 2013 to 2018 NHANES. A total of 26,026 individuals remained after satisfying inclusion and exclusion criteria.

A total of 5,432 subjects were included in this analysis after excluding males (*n* = 14,452) and those with missing prevalence of PID (*n* = 9,516) (Figure 1). Age at participation was 38.1 ± 12.4 years (Table 1). The Depression Score level of 314 PID patients in the 2013–2018 NHANES was 6.5 (6.3) 4.0 (0.0–25.0), Mean (SD) Median (Min-Max)/N (%). Patients with PID had a significantly higher Depression score than those with Non-PID (Table 1).

**TABLE 1:** Characteristics of participants (*N* = 5432).

Prevalence of PID	Overall (*n* = 5432)	Non-PID (*n* = 5118)	PID (*n* = 314)	Standardize diff. (CI)	*P*-value	*P*-value*
Age (years, mean ± SD)	38.1 ± 12.4	37.8 ± 12.4	42.3 ± 11.2	0.4 (0.3, 0.5)	< 0.001	< 0.001
Race *n* (%)				0.3 (0.1, 0.4)	< 0.001	−
Non-Hispanic White	1809 (33.3%)	1697 (33.2%)	112 (35.7%)			
Mexican American Other Hispanic	1498 (27.6%)	1439 (28.1%)	59 (18.8%)			
Non-Hispanic Asian Other race	912 (16.8%)	864 (16.9%)	48 (15.3%)			
Non-Hispanic Black	1213 (22.3%)	1118 (21.8%)	95 (30.3%)			
Education *n* (%)				0.2 (0.1, 0.2)	< 0.001	
High school below	837 (15.4%)	520 (13.6%)	317 (19.8%)			
High school graduate or above	4595 (84.6%)	3312 (86.4%)	1283 (80.2%)			
Depression score	3.8 (4.6)	3.6 (4.4)	6.5(6.3)	0.5(0.4,0.7)	< 0.001	< 0.001
Mean (SD) Median (Min-Max)/N (%)	2.0 (0.0-27.0)	2.0 (0.0-27–0)	4.0(0.0,25.0)			
Depression severity				0.5 (0.4, 0.6)	< 0.001	−
None	3421(63.0%)	3281 (64.1%)	140 (44.6%)			
Mild	1285 (23.7%)	1205 (23.5%)	80 (25.5%)			
Moderate	445 (8.2%)	401 (7.8%)	44 (14.0%)			
Moderately severe	185 (3.4%)	156 (3.0%)	29 (9.2%)			
Severe	96 (1.8%)	75 (1.5%)	21 (6.7%)			
Regular menstruation				0.2 (0.1, 0.2)	< 0.001	
No	1599 (29.4%)	1039 (27.1%)	560 (35.0%)			
Yes	3833 (70.6%)	2793 (72.9%)	1040 (65.0%)			
BMI (mean ± SD)	29.9 ± 8.3	29.8 ± 8.3	31.3 ± 8.8	0.2 (0.1, 0.3)	0.002	0.002
WBC	7.6 ± 2.3	7.6 ± 2.3	7.6 ± 2.5	0.0 (−0.1, 0.1)	0.967	0.698
Segmented neutrophils	4.5 ± 1.8	4.5 ± 1.8	4.4 ± 2.1	0.0 (−0.1, 0.1)	0.652	0.194

CI, confidence interval, OR, odds ratio; PID, pelvic inflammatory disease; WBC, white blood cell count (1,000 cells/ul); BMI, body mass index (1,000 cell/uL, mean ± SD). *P*-value: Obtained by t-test or chi-square test. *P*-value*: For continuous variables, Kruskal–Wallis rank test, Fisher exact for categorical variables, with expects <10.

### Univariate analysis of prevalence of pelvic inflammatory diseases

The increase in age was significantly correlated with the occurrence of prevalence of PID (OR1.03, 95%CI 1.02–1.04). Further, women with irregular menstrual cycles had a lower risk of developing prevalence of PID (OR 0.51, 95%CI 0.40–0.64; Table 2).

**TABLE 2:** Univariate analysis for prevalence of pelvic inflammatory diseases.

Covariate	Statistics	OR (95% CI)	*P*-value
AGE (years, mean ± SD)	38.09 ± 12.36	1.03 (1.02, 1.04)	< 0.0001
**RACE *n* (%)**			
Non-Hispanic white	1809 (33.30%)	Ref	
Mexican American Other Hispanic	1498 (27.58%)	0.62 (0.45, 0.86)	0.0039
Non-Hispanic Asian Other race	912 (16.79%)	0.84 (0.59, 1.19)	0.3318
Non-Hispanic black	1213 (22.33%)	1.29 (0.97, 1.71)	0.0808
**EDUCATION *n* (%)**			
high school below	837 (15.41%)	Ref	
High school graduate or above	4595 (84.59%)	0.83 (0.62, 1.12)	0.2206
**Regular menstruation**			
No	1599 (29.44%)	Ref	
Yes	3833 (70.56%)	0.51 (0.40, 0.64)	< 0.0001
BMI (mean ± SD)	29.89 ± 8.35	1.02 (1.01, 1.03)	0.0020
Depression score	3.75 ± 4.59	1.10 (1.08, 1.12)	< 0.0001
**Depression severity**			
None	3421 (62.98%)	Ref	
Mild	1285 (23.66%)	1.56 (1.17, 2.06)	0.0022
Moderate	445 (8.19%)	2.57 (1.80, 3.66)	< 0.0001
Moderately severe	185 (3.41%)	4.36 (2.83, 6.70)	< 0.0001
Severe	96 (1.77%)	6.56 (3.93, 10.96)	< 0.0001
WBC	7.57 ± 2.30	1.00 (0.95, 1.05)	0.9668
SN	4.46 ± 1.83	0.99 (0.92, 1.05)	0.6516

CI, confidence interval; OR, odds ratio; PID, Pelvic inflammatory disease; WBC, White blood cell count (1,000 cells/ul); BMI, body mass index; SN, Segmented neutrophil.

### Associations between prevalence of pelvic inflammatory diseases and the depression score

**FIGURE 2:**
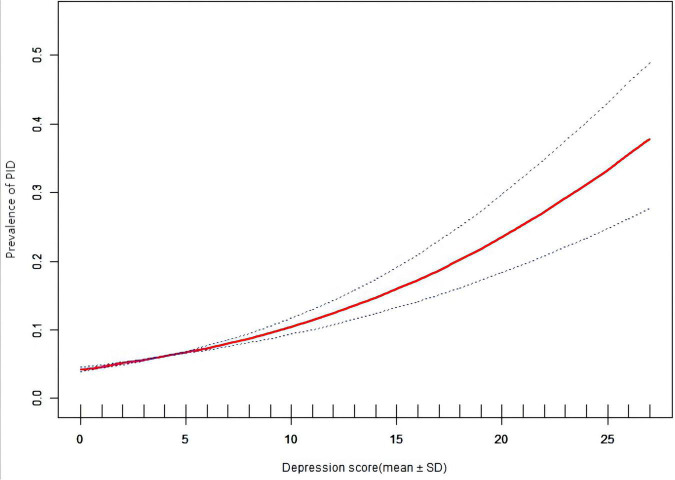
Prevalence of pelvic inflammatory diseases (PID) vs. depression score: association PID and depression score: A linear association between PID and depression score was found in a generalized additive model (GAM). The solid red line represents the smooth curve fit between variables. Blue bands represent the 95% of confidence interval from the fit. All adjusted for age, education, race, BMI, Regular menstruation, WBC, Segmented neutrophils.

After further adjustment for age, race/ethnicity, education, body mass index, menstrual cycle, white blood cell, segmental, neutrophils factors. In multiple logistic regression analysis and curve fitting, we found that there was a significant correlation between depression index and the prevalence of PID. A multifactor regression analysis revealed a continuous relationship between prevalence of PID and Depression (Figure 2 and Table 3).

**TABLE 3:** Relationship between depression severity and prevalence of pelvic inflammatory diseases.

Outcome	Crude model		Model I		Model II	
	OR (95% CI)	*P*-value	OR (95% CI)	*P*-value	OR (95% CI)	*P*-value
Depression score (mean ± SD)	1.10 (1.08, 1.12)	< 0.0001	1.10 (1.08, 1.12)	< 0.001	1.10 (1.08, 1.12)	< 0.0001
**Depression-severity *n* (%)**						
None	Ref		Ref		Ref	
Mild	1.56 (1.17, 2.06)	0.0022	1.48 (1.11, 1.97)	0.0081	1.50 (1.12, 2.00)	0.0060
Moderate	2.57 (1.80, 3.66)	< 0.0001	2.33 (1.62, 3.35)	< 0.0001	2.35 (1.64, 3.38)	< 0.0001
Moderately Severe	4.36 (2.83, 6.70)	< 0.0001	4.01 (2.57, 6.27)	< 0.0001	4.01 (2.56, 6.28)	< 0.0001
Severe	6.56(3.93,10.96)	< 0.0001	6.36 (3.74, 10.82)	< 0.0001	6.34(3.72,10.79)	< 0.0001

CI, confidence interval. Model I adjusted for age, education, and race. Model II adjusted for age, education, race, BMI, Regular menstruation, WBC, Segmented neutrophils.

### Association between prevalence of pelvic inflammatory diseases and the depression severity

**FIGURE 3:**
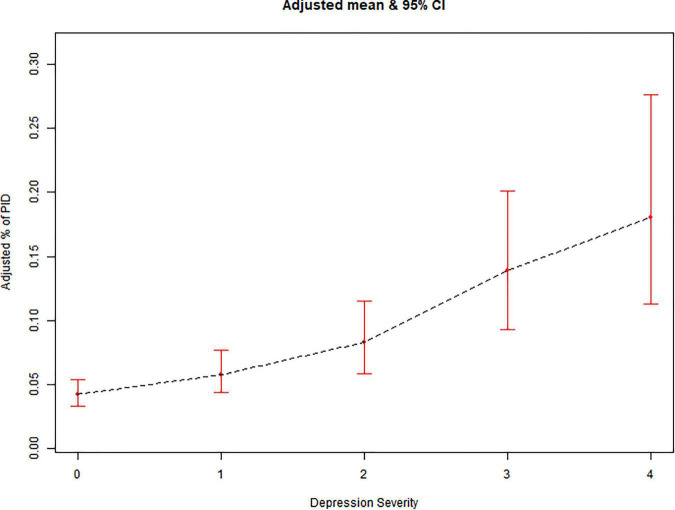
Prevalence of pelvic inflammatory diseases (PID) vs. depression severity: association PID and depression severity: A linear association between PID and Depression Severity was found in a generalized additive model (GAM). 0 = Non, 1 = Mild, 2 = Moderate, 3 = Moderately Severe, 4 = Severe.

Results of multivariate logistic regression analysis and curve fitting showed a correlation between the prevalence of PID and depression (Figure 2). Multivariate logistic regression analysis and curve fitting revealed that prevalence of PID risk increased with depression severity. Age, race/nationality, education, body mass index, menstrual cycle, white blood cells and segmented neutrophils as risk factors for PID were included in the study as adjustment factors. The results of multivariate Logistic regression analysis and curve fitting showed that the prevalence of mild depression was higher than that of non-depression (OR 1.50, 95%CI 1.12–2.00). In addition, with the increase of depression index and the aggravation of depression, the correlation between prevalence of PID and depression may be more significant (OR 6.34, 95%CI 3.72–10.79). There was a positive correlation between the degree of depression and the prevalence of PID. This conclusion is also supported by the linear relationship in the smooth curve fitting of the two (Figure 3).

**FIGURE 4:**
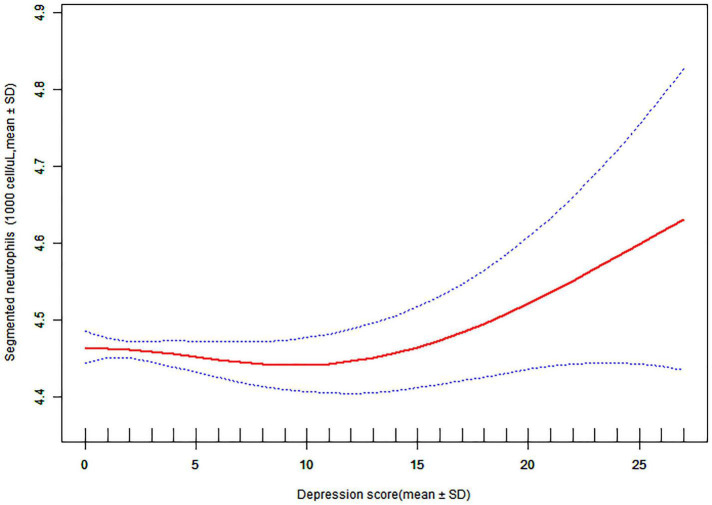
Association depression score and segmented neutrophils: A threshold, non-linear association between depression severity and segmented neutrophils were found in a generalized additive model (GAM).

It is worth noting that in the smooth curve fitting analysis, the non-linear correlation between the subjects with different depression index and their segmented neutrophil count was observed: when the depression index was higher than a numeric value, the higher the depression index was, the higher the segmented neutrophil count in blood was, and there was a positive correlation between them. Unfortunately, we did not calculate the turning value of the depression index, this is also the focus of our further research on the relationship between the two in the future. That may partly explain why people with depression have such a high risk of prevalence of PID (Figure 4).

### Subgroup analysis

**FIGURE 5:**
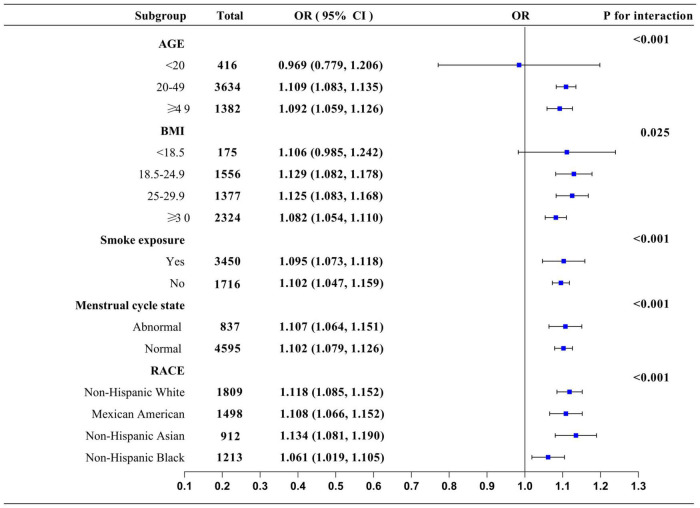
Subgroup analysis: Forest plot showing the OR and 95%CI of incidence of pelvic inflammatory diseases risk in patients with different degrees of depression in different subgroups.

We used subgroup analysis and interaction testing to further understand this outcome. The age range was separated into three categories: under 20, 20–49, and above 49. According to WHO guidelines, BMI was categorized as 18.5, 18.5–24.9, 25.0–29.9, and 30.0 kg/m^2^. This study determined if the association between prevalence of PID and the Depression Score still applied to each subgroup based on age, gender, race, BMI, menstrual cycle status, and smoking exposure. There was an interaction between prevalence of PID and the Depression Score across menstrual cycles, age, BMI, and racial groups, according to the findings. We found a greater correlation between prevalence of PID and the Depression Score in the population of women of reproductive age between 20 and 49 years of age compared to patients younger than 20 years of age and those older than 49 years of age, with an effect value and 95% confidence interval of (OR 1.109, 95% CI 1.083–1.135). When compared to other ethnic groups, depression was more linked with the prevalence of pelvic inflammatory disease in the Non-Hispanic-Asian population, with an effect value and 95% confidence interval of (OR 1.134, 95% CI 1.081–1.190). There was no significant difference between prevalence of PID and Depression Score in smokers versus non-smokers (Figure 5).

## Discussion

With the increase in depression score and the aggravation of Depression severity, we found that the prevalence of PID was not significantly correlated with BMI, race, and education level. When compared with the non-Depression group, the OR of the Severe-Depression group increased the most (OR 6.43, 95%CI 3.72–10.79), followed by the Moderately Severe group (OR 4.01, 95%CI 2.56–6.28) and the Moderate group (OR 2.35 95%CI 1.64–3.38). The prevalence of PID increased 6.34 times, 4.01 times, and 2.35 times, respectively.

In addition, we also analyzed the potential mechanism of depression scores on the occurrence of PID in women. For every 1 increase in depression scores, the risk of pelvic inflammation was 1.10 times higher than before. Interestingly, although there was no difference between WBC and Segmented neutrophils in the prevalence of PID group and Non-PID Group, we found a non-linear relationship between them in smooth curve fitting between Segmented neutrophils and Depression Severity: when the depression score was less than a numeric value, there was no correlation between the depression index and the serum Segmented neutrophils equivalent, but when the depression index was more than numeric value and gradually increased, the Segmented neutrophils equivalent increased, and there was a positive correlation between them. In previous studies, there has been a close relationship between depression and inflammatory factors. A persistently activated hypothalamus-pituitary adrenal (HPA) axis combined with excessive cortisol release impair the sensitivity of glucocorticoid receptors (GR) and increases the activity of the pro-inflammatory immune response. It may be inferred that the serum segmented neutrophils equivalent will increase abnormally if the severity of depression is increased.

A series of symptoms are often accompanied by PID, such as abnormal leucorrhea, fever, and abdominal pain, which can be completely cured when the disease is treated with antibiotic. Pelvic inflammation caused by low autoimmunity and drug resistance is often accompanied by intermittent abdominal pain and low back pain in patients. Chronic pain and depression are very common diseases, and one disease increases the risk of the other. An array of physiological and behavioral processes is involved in depression and pain. Under chronic stressful conditions, or even after a stressful event, the hypothalamic-pituitary-adrenal (HPA) axis can be overactivated or activated. A variety of antidepressants have been proven to relieve chronic pain in patients (12). In comparison with healthy controls, depression patients display an up-regulation of inflammation markers in peripheral and central nervous system tissues, including elevated concentrations of pro-inflammatory cytokines and immune mediators in cerebrospinal fluid (13). However, there is little evidence that there is a correlation between depression and PID, and there is little literature on the correlation between the severity of depression and PID. Our results are supported by data that show that the correlation between the severity of depression and PID is biologically reasonable. Due to the secondary nature of the analysis, we are unable to collect new data, which may result in residual confusion in the unmeasurable covariates. For the lack of serum C-reactive protein (CRP) data, we choose to ignore this covariate for analysis. In addition, limited information is available regarding the treatment, prognosis, and duration of specific PID. In this study, the population is large and representative. Severity and outcome variables of depression, as well as PID, were also assessed in a standardized manner.

Our research has a number of advantages. Firstly, a large sample of public databases was applied, which enhances the authority and reliability of the data. Secondly, the application of multi-factor regression analysis allows for the inclusion of more risk factors in the analysis, which enhances the credibility of the results, and finally, the relatively large sample size allows for more objective conclusions.

Inevitably, there are some limitations to our study. Firstly, cross-sectional analysis can only examine the distribution of risk factors for PID in a specific population at a specific time, and the study population is limited to adult women in the USA.

## Conclusion

According to this study, depression severity among women in the US was associated with pelvic inflammatory disease. Even after adjusting for possible confounding factors, this correlation persists. Diagnosis and treatment of concomitant depression should be strongly considered in patients with PID.

## Data availability statement

The raw data supporting the conclusions of this article will be made available by the authors, without undue reservation.

## Ethics statement

The studies involving human participants were reviewed and approved by National Health and Nutrition Examination Survey (NHANES). The patients/participants provided their written informed consent to participate in this study. Written informed consent was obtained from the individual(s) for the publication of any potentially identifiable images or data included in this article.

## Author contributions

XY and JL obtained funding. TH and RC designed the study. TH and PL collected the data, involved in data cleaning, mortality follow-up, and verification. TH and XY analyzed the data. RC and TH drafted the manuscript. All authors contributed to the interpretation of the results and critical revision of the manuscript for important intellectual content and approved the final version of the manuscript and have read and approved the final manuscript.

## Footnotes

1
http://www.R-project.org
